# Life of Pi: Exploring functions of *Pi16*+ fibroblasts

**DOI:** 10.12688/f1000research.143511.2

**Published:** 2024-07-08

**Authors:** Erika E. McCartney, Yein Chung, Matthew B. Buechler

**Affiliations:** 1 Department of Immunology, University of Toronto, Toronto, Ontario, M5S1A8, Canada

**Keywords:** fibroblast, Pi16

## Abstract

Fibroblasts are mesenchymal cells that are responsible for creating and maintaining tissue architecture through the production of extracellular matrix. These cells also play critical roles in processes such as wound repair and immune modulation in normal tissues and various disease states including fibrosis, autoimmunity, and cancer. Fibroblasts have a complex repertoire of functions that vary by organ, inflammatory state, and the developmental stage of an organism. How fibroblasts manage so many functions in such a context-dependent manner represents a gap in our understanding of these cells. One possibility is that a tissue-resident precursor cell state exists that provides the fibroblast lineage with flexibility during growth, inflammation, or other contexts that require dynamic tissue changes. Recent work has suggested that a precursor fibroblast cell state is marked by expression of *Peptidase inhibitor 16* ( *Pi16*). This review aims to concatenate and compare studies on fibroblasts that express *Pi16* to clarify the roles of this cell state in fibroblast lineage development and other functions.

## 1. Introduction – On site fibroblast precursors

Cells of the fibroblast lineage are found across the entire body, where they maintain tissue architecture through the production of extracellular matrix, interact with immune cells, and contribute to a wide range of pathologies such as fibrosis, cancer, and autoimmunity. ^1^ Understanding how this functional heterogeneity occurs and its role in immune homeostasis and inflammation has been a challenge. Parabiosis experiments suggest that fibroblasts that arise during inflammation are seeded from local, *in situ* local precursors, indicating that a circulating progenitor cell is not a likely source of new fibroblasts in tissue. ^2^^,^^3^ One means by which fibroblasts may achieve parallel generalized function such as ECM remodeling, while also having context-specific phenotypes is via a precursor cell that resides within tissues. In this conceptual model, a fibroblast cell state exists that can develop into more specialized fibroblast subtypes based on context specific signalling, providing this essential cell lineage flexibility during development and disease conditions.

In 2021, Turley and colleagues used a combination of 28 publicly available mouse fibroblast single cell RNA sequencing (scRNAseq) datasets to create a fibroblast transcriptional atlas to address this question. They identified that cells of the fibroblast lineage could be divided into either context-specific clusters that were influenced by tissue (e.g., *Ccl19*+ fibroblastic reticular cells in secondary lymphoid organs) or clusters that were found in all tissues (lymph node, omentum, thymus, lung, skin [ear and flank], adipose [epidydimal, subcutaneous, and brown], pancreas, and heart). ^4^ These conserved clusters expressed *Dermatopontin* ( *Dpt*) and could be further characterized by their expression of *Peptidase inhibitor 16* ( *Pi16*) and *Collagen 15a1* ( *Col15a1*). The existence of these subsets in the aforementioned tissues was validated *in vivo* using a *Dpt.*IRES.cre.ERT2; *Rosa26.*Lox-Stop-Lox.YFP (i *Dpt* ^YFP^) reporter mouse. ^4^

The role of cell states in biology can be challenging to unequivocally demonstrate as laboratories use different techniques, employ distinct nomenclatures, and examine different tissues or contexts. Cells that transcriptionally match *Pi16*+ fibroblasts have been termed universal fibroblasts, ^4^ adventitial fibroblasts, ^5^ interstitial progenitor cells, ^6^ multipotent progenitor cells, ^7^ fibro-adipogenic progenitors, ^8^ and fibro-inflammatory progenitors. ^9^^,^^10^ *In silico* analyses have revealed a list of marker genes that are expressed by *Pi16+* fibroblasts, including *Dpp4* (Dipeptidyl peptidase-4), *Cd55, Ackr3* (atypical chemokine receptor, encodes CXCR7), *Anxa3* (Annexin A3) *,* and *Il33* (See Table 1).

**Table 1.** The role of cell states in biology can be challenging to unequivocally demonstrate as laboratories use different techniques, employ distinct nomenclatures and examine different tissues or contexts.

Cell name	Enriched genes	Tissue(s)	Group
Adventitial stromal cells	*Pi16, Ccl11, Cd34, Cxcl1, Il33, Ly6a,*	Lung, liver, subcutaneous adipose, visceral adipose	Dahlgren *et al.*, 2020
Fibro-adipogenic progenitors	*Pi16, Dact2, Sfrp4, Wnt2, Sema3c, Tgfrbr2*	Skeletal Muscle	Scott *et al.*, 2019
Fibroinflammatory progenitors	*Pi16, Dpp4, Ccl2, Fn1, Loxl2, Ly6C1, Tgfb2*	Visceral adipose, subcutaneous adipose	Helper *et al.*, 2018; Shao *et al.*, 2021
Interstitial progenitor cells	*Pi16, Dpp4, Bmp7, Cd34, Ly6a, Thy1, Wnt2*	Subcutaneous adipose	Merrick *et al.*, 2019
Multipotent progenitor cells	*Pi16, Dpp4, Anxa3, Cd55, Dpt*	Visceral adipose	Kabat *et al.*, 2022
Stromal mesenchymal progenitors	*Aldh1a2, Hic1, Hmga2, Id1, Osr1, Snai1, Sox11, Tcf7l2*	Skeletal Muscle	Arostegui *et al.*, 2022
Universal fibroblasts	*Pi16, Dpp4, Cd34, Dpt, Ly6c1*	Lymph node, omentum, lung, ear skin, flank skin, visceral adipose, subcutaneous, adipose, brown adipose, pancreas, and heart	Buechler and Prahdan *et al.*, 2021

The presence of transcriptionally analogous fibroblast states across the body may suggest a *Pi16+* fibroblast niche that exists across tissues. *Pi16+* fibroblasts have been identified near vasculature numerous tissues including the lung, ^4^ tonsil, ^11^ muscle, ^8^^,^^12^ and mammary gland, ^13^ and near neural structures in the muscle ^12^ and central nervous system. ^14^ Notably, fibroblasts with a *Pi16+* phenotype were also found away from blood vessels, instead associated with the reticular interstitium, ^15^ a band of fluid-filled space enmeshed with fibrous collagen found within and across tissues including skin and subcutaneous adipose. ^6^^,^^16^ These shared niches may either suggest a conserved function across tissues or may be the driving force in maintaining transcriptional similarity across tissues.

## 2. Lineage functions of the *Pi16+* fibroblast

Our understanding of fibroblast biology has expanded greatly since the development of scRNAseq. This approach uncovered distinct functional fibroblast subsets within a single tissue. It was *in silico* trajectory analysis, ^4^ since performed by numerous groups, ^14^^,^^16^^,^^17^ that first suggested a lineage relationship in which *Pi16-*expressing cells give rise to the various tissue specific fibroblast subsets, possibly through a *Col15a1+* intermediate cell state. It is worth noting that in several instances, the *Pi16+* fibroblast cluster was set as the initial root cluster during lineage inference. ^4^^,^^13^^,^^16^^,^^17^

Empirical validation of *in silico* approaches that suggest a lineage relationship amongst fibroblasts beginning at the *Pi16+* stage has been examined within adipose tissue. Adipogenesis is the process by which adipocytes, key cells for energy storage, develop. This can occur via two processes: hyperplasia, the development of adipocytes from a precursor cell, and hypertrophy, the process by which adipocytes increase in size to accommodate increased lipid storage. The contribution of fibroblasts to adipogenesis via hyperplasia was first confirmed using genetic tools *in vivo* in 2013 when adipocytes in white adipose tissue were shown to be derived from Platelet-derived growth factor alpha expressing (PDGFRa+) cells using a *Pdgfra.*cre mouse. ^18^ *In vivo* adipogenesis is complex and may exhibit temporal contours. ^19^^,^^20^ Current models suggest early adipogenesis is dependent on Peroxisome proliferator-activated receptor gamma ( *Pparg*) in PDGFRA-expressing cells. ^21^ However, in later-stage adipogenesis, non-fibroblastic mural cells may contribute to the adipocyte pool. ^20^

A study by Merrick *et al.,* described two fibroblast populations within white adipose tissue that contribute to adipogenesis in a stepwise fashion. One population was defined by *Pi16*, *Cd34*, and CD26 (encoded by *Dpp4*) *,* while the other, a preadipocyte was marked by ICAM1 and shares attributes with *Dpt+Col15a1+* population identified by Buechler and Pradhan *et al*. ^4^^,^^22^ In *in vitro* adipogenesis assays, both populations of fibroblasts exhibited adipogenic capacity. The ICAM1+ preadipocytes gave rise to adipocytes faster and in greater numbers than the *Pi16*+ populations. This discrepancy in kinetics was attributed to the preadipocytes being a more differentiated cell state. ^22^ To assess their *in vivo* contributions to adipogenesis, *Pi16+* and ICAM1+ cells isolated from congenically-marked (TdTomato) mice were transplanted into adipose depots of recipient mice. Transplantation of *Pi16+*-like cells resulted in TdTomato+ ICAM+ preadipocytes as well as mature adipocytes, while transplant of ICAM1+ cells resulted only in mature adipocytes. ^22^ This elegant cell transfer system confirmed that *Pi16+* cells can function as a precursor for the adipocyte lineage and that differentiation occurs in a stepwise manner from through an ICAM1+ ( *Col15a1+*) intermediate *in vivo.*

Yet, these results did not confirm the *in situ* role of *Pi16+* cells *in vivo* within adipose tissue. To this end, the same group used a *Dpp4.*creERT2;GFP mouse line, to reveal that CD26+ cells contributed to mature adipocytes when mice were on both a normal chow or high fat diet (HFD). ^23^ Mice on HFD also exhibited a greater number of GFP+ adipocytes after 24 weeks. This mouse model also confirmed the finding that CD26+ cells give rise to ICAM1+/ *Col15a1+* preadipocytes *in vivo.*

Contexts outside the adipogenesis field also indicate that *Pi16+* fibroblasts may function as a precursor cell for the fibroblast lineage. The Underhill and Rossi groups proposed that *Hypermethylated in cancer 1* ( *Hic1*) marked tissue resident mesenchymal progenitors. The transcriptional definition of *Hic1*+ cells, *Pdgfra, Thy1,* and *Gli1,* suggests these cells may represent *Pi16+* cells in the muscle. Pulse-chase experiments in the heart using a *Hic1* ^creERT2^;Rosa26 ^TdTomato^ mouse, which specifically labels *Hic1+* fibroblasts with TdTomato, demonstrated that a small proportion of specialized cardiac fibroblasts were TdTomato+ at both 7 and 35 days post-pulse. ^24^ This suggests that fibroblasts which share phenotypic characteristics with *Pi16+* fibroblasts contribute to specialized fibroblast subsets at steady state in the heart.

Interestingly, *Hic1* may also functionally regulate fibroblast biology *in vivo.* Cells that exhibit the phenotype of *Pi16+* fibroblasts were shown to be in a state of quiescence in the steady state, with minimal proliferation in uninjured tissues. ^8^ The ubiquitous deletion of *Hic1* using UBC.cre.ERT2; *Hic1*flox/flox mice, led to the rapid expansion of fibroblasts, quantified by flow cytometry and the incorporation of EdU (5-ethynyl-20-deoxyuridine) in the *Pi16+*-like population. ^8^ The deletion of *Hic1* also had an activating effect on these cells – comparison of differentially expressed genes showed a substantial overlap of genes with injury-activated *Hic1+ cells.* ^8^ The deletion of *Hic1* in *Pdgfra+* fibroblasts ( *Pdgfra.*CT2; *Hic1*Flox/Flox mouse) resulted in epicardium fibrosis and accumulation of adipocytes that drastically reduced cardiac function. ^24^ This study not only suggests *Hic1* is required for the quiescence of *Pi16+-*like fibroblasts but demonstrates the multipotent capacity of these cells.

The quiescence of *Pi16+* cells, which can be unlocked by loss of *Hic1* using genetic tools, ^24^ may have physiological relevance. In the spared nerve injury (SNI) model of chronic neuropathic pain, *Pi16* expression was increased in both the sciatic nerve and lumbar dorsal root ganglia 8 days post-injury compared to sham surgery controls. An increased number of *Pi16+* fibroblasts were also demonstrated in proximity to the injury site. ^14^ This suggests that while *Pi16+* fibroblasts are mitotically inactive in healthy tissues, expansion may be induced by damage to facilitate tissue repair.

Buechler and Pradhan *et al.,* observed *Pi16+* fibroblasts across diseased tissues in mice and humans. Transcriptionally, *Pi16+* fibroblasts retained expression of genes associated with stemness during inflammation and trajectory analysis maintained the lineage relationship from *Pi16+* to *Col15a1+* before differentiation into specialized tissue-specific fibroblasts or pathological *Lrrc15*+ myofibroblasts. ^4^ *In silico* trajectory analysis of fibroblast clusters within a dorsal skin wound validated this concept in the skin, showing that *Pi16+* fibroblasts within the fascia adopt a proinflammatory state before differentiating into myofibroblasts. ^16^ The differentiation of fibroblasts with a *Pi16+* phenotype to myofibroblasts in wound repair was shown to be required as blocking this differentiation resulted in weakened and poorly organized ECM fibers, and poor wound closure. ^16^

Lineage tracing has confirmed that *in situ* fibroblasts may act as precursor cells to more specialized fibroblasts. Using the i *Dpt* ^YFP^ mouse, Turley and colleagues showed in two studies that in subcutaneous and orthotopic models of pancreatic cancer LRRC15+ myofibroblasts derive from *Dpt+* fibroblasts. ^4^^,^^25^ A study by Houthuijzen *et al*. found that different subsets of cancer associated fibroblasts (CAFs) in breast cancer are derived from CD26+ and CD26- fibroblasts within the mammary gland. ^26^ CD26- fibroblasts gave rise to myofibroblastic CAFs, while CD26+ cells preferentially produced inflammatory CAFs. Despite this, CD26+ CAFs also showed features in line with myofibroblastic CAFs, suggesting that either inflammatory CAFs are functionally plastic and can contribute to both inflammatory processes and myofibroblastic functions or myofibroblasts may develop directly from a CD26+ intermediate. Lineage tracing experiments using a tool such as the *Dpp4.*creERT2;GFP mouseline ^23^ would be helpful to confirm the contribution of CD26+ *Pi16+* cells to these CAF phenotypes.

The contribution of *Pi16+* cells to specialized fibroblasts and adipocytes has been demonstrated in various tissues at steady state as well as in numerous disease contexts and injury models, indicating a progenitor role for context-specific fibroblast subsets. The ability of these cells to generate numerous cell states within the same tissue, as seen upon *Hic1* deletion in the heart ^24^ and by contribution to distinct CAF subsets in breast cancer, ^26^ affirms the multipotent capacities of these cells *in vivo.*

## 3. Non-lineage functions of the *Pi16+* fibroblast

The concept of a fibroblastic stem-like cell marked by *Pi16* that resides across tissues to give rise to other fibroblast cell types is simple and conceptually attractive. Yet, if *Pi16* marks a fibroblast lineage, a concept that has not yet been proven, the function of these cells *in vivo* may be distinct from a strictly precursor role. Alternatively, these cells may serve several functions, either due to inherent plasticity, heterogeneity within *Pi16+* cells, temporal regulation during development, or other factors. Indeed, some studies have suggested a non-precursor role for *Pi16+* cells.

Arostegui *et al.* described an embryonic population of mesenchymal progenitor cells marked by *Hic1* in the limb bud that persist into adulthood. These cells were shown to overlap transcriptionally with *Pi16+* fibroblasts. ^12^ In this study, *Hic1+* cells appeared in the limb bud at embryonic day 11.5 (E11.5) and these cells co-localized with CD31+ endothelial cells, consistent with other studies. ^4^^,^^12^^,^^14^ ScRNAseq of *Hic1*-labeled cells from E11.5-E16.5 suggested that these cells operate as a precursor population in the developing limb bud. Cells from earlier time points (E11.5 and E12.5) clustered together and were transcriptionally distinct from *Hic1*-labeled cells at E14.5 and E16.5. The E11.5+12.5 cluster expressed genes associated with a progenitor phenotype, while E14.5+E16.5 cells formed clusters suggestive of several distinct differentiated phenotypes, including numerous fibroblast subsets, chondrocytes, and pericytes. ^12^ Trajectory analysis of this data set suggests a direct lineage relationship between *Hic1+* cells and each of the differentiated phenotypes identified at E14.5 and E16.5. A pulse of tamoxifen at E10, labelling cells currently expressing *Hic1+* with TdTomato, allowed for *in vivo* validation of this finding. By E18.5, *Hic1+* cells contributed to chondrocytes, tenocytes, pericytes, and numerous fibroblast subsets, as visualized by immunofluorescence. ^12^ This indicates that *Hic1+* cells are a functional and multipotent progenitor of numerous mesenchymal cell types during embryonic limb development. Interestingly, within the fibroblasts marked by the *Hic1* allele, a bifurcation existed at E18.5 between *Pi16+* cells (Fibroblast I) and other fibroblasts (Fibroblast II), suggesting that *Pi16+* cells may represent a distinct, non-precursor lineage. Consistent with this finding, in developing lymph nodes, *Pi16+* fibroblasts were identified *in silico* but were suggested to not operate as a stem-like reservoir population. ^27^ However, the contours of *Pi16*+ fibroblast development, and that of the entire fibroblast lineage, across tissues and timepoints during embryogenesis remains largely unexplored.

In the context of skin, Rinkevich *et al.* described a population of cells marked by the gene Engrailed-1 ( *En1*). ^28^ The *En1.*cre allele first emerges in the skin at embryonic day 14.5 (E14.5). ^29^ The cells, named *En1* positive fibroblasts (EPFs), become the dominant fibroblast population by post-natal day 0 (P0). ^29^ Similar to *Pi16+* cells as described by Buechler and Prahdan *et al.* the EPFs were marked by CD26 ( *Dpp4*) expression and were the dominant fibroblast type in the adult skin. ^4^^,^^28^ EPFs directly contributed to skin architecture maintenance and wound repair through the production of collagen. In a later study by Correa-Gallegos *et al.* EPFs within the skin fascia were described as expressing both *Dpt* and *Pi16.* ^16^ Interestingly, the inhibition of CD26 in the skin led to delayed wound healing and decreased scar size, ^28^ which may suggest a crucial role of CD26 in the activation and/or function of *Pi16+* cells.

*Pi16+* fibroblasts may also be pro-fibrotic; Gupta and colleagues identified fibro-inflammatory progenitors (FIPs) using a *Pdgfrb* ^rtTA^;TRE-cre;Rosa26R ^mT/mG^ “MuralChaser” mouse in both visceral and subcutaneous adipose depots by scRNAseq. ^30^ FIPs were shown to exhibit the highest expression of extracellular matrix transcripts suggestive of a pro-fibrotic function. ^30^^,^^31^ FIPs also exhibit some potential to develop into adipocytes, suggesting dual roles as progenitors and key players in fibrosis, possibly due to heterogeneity within this population. ^9^ Analysis in the murine lung has supported the concept that some *Pi16+* cells may be stem-like whereas other *Pi16+* cells adopt roles during fibrosis. ^32^

*Pi16* expressing fibroblasts have been identified in numerous secondary lymphoid organs across species. These cells have similar transcriptional profiles across tissues but exhibit some tissue level imprinting as defined by scRNAseq. Cell-cell interactions were predicted using *in silico* approaches and suggested *Pi16+* cells produced a variety of chemokines and cytokines which likely have a pro-inflammatory effect on both myeloid cells and lymphocytes. ^11^^,^^33^ Ludewig and colleagues identified *PI16+* fibroblasts localized to vessel rich regions of the human tonsil and make direct contact with B cells and T cells. ^11^ *In vitro* assays confirmed that these tonsillar *Pi16+* fibroblasts were potent mediators of T cell activation. ^11^ In the muscle injury model, *Hic1+* fibroblasts cells co-cultured with activated CD3+ T cells demonstrated that *Pi16+* fibroblasts induce significant proliferation in both CD4+ and CD8+ T cells. CD25, a marker of T cell activation, was also seen to increase in response to co-culture with *Pi16+* cells in comparison to stimulation with PHA alone. ^14^

The presence of *Pi16+* fibroblasts in a shared niches across tissues may suggest a conserved function across the body. *Pi16+* fibroblasts may mediate extravasation of cells into tissues. ^12^^,^^14^ Their direct role in this process is not understood but may be through enzymatic activity of *Pi16* itself or CD26, which are both known to modify proteins associated with immune cell trafficking. ^34^^,^^35^ *Pi16* knockout mice showed a protection from pain response after SNI, decreased leukocyte infiltration into the dorsal root ganglia, as well as decreased endothelial permeability. ^14^ Depletion of fibroblastic progenitor cells in the muscle showed a similar decrease in immune cell accumulation post-injury. ^8^ This suggests *Pi16+* cells play a role in immune infiltration upon injury, which is consistent with their proximity to vessels across tissues and the enzymatic functions enzymatic activity of *Pi16* and CD26 *,* which are both known to modify proteins associated with immune cell trafficking. ^34^^,^^35^ If *Pi16+* fibroblasts are required for extravasation and immune infiltration, therapeutic exploitation of these pathways in the tumour microenvironment could be used to modify cold tumours and immune deserts into immune rich tumors. ^1^^,^^8^

## Conclusions

**Figure 1.**
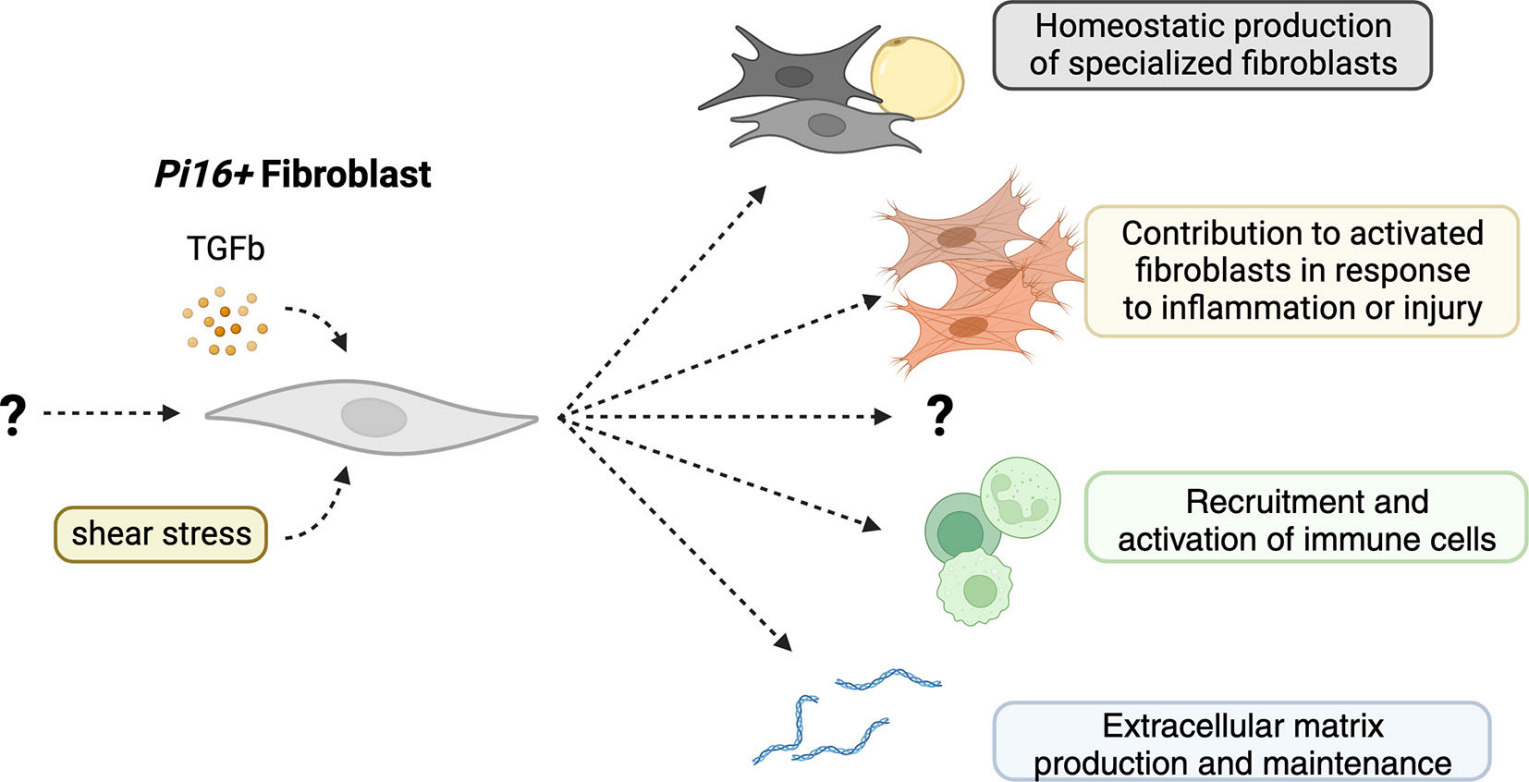
Possible functions of *Pi16+* fibroblasts *in vivo*. *Pi16* expressing fibroblasts, driven by TGF-β and shear stress, contribute to the production of specialized fibroblasts at homeostasis and myofibroblasts in inflammation and injury. They also may contribute to the production of extracellular matrix and the recruitment and activation of immune cells through the production of inflammatory cytokines.

*Pi16+* fibroblasts have been found across tissues with similar gene expression profiles and localization patterns through a combination of *in vivo* and *in silico* approaches. In some contexts, *Pi16+* fibroblast-like cells have exhibited the potential to act as a precursor cell for fibroblasts and fibroblast-derived cells, including but not limited to myofibroblasts, adipocytes, and CAFs ( Figure 1). These cells are seen across the body at both steady state and in many disease models, suggesting a functional importance in all contexts. Further characterization of these cells across tissues and contexts, however, is important to confirm each of these studies is truly describing the same cell state. The epigenetic landscape of these cells has also not been described, which may provide important insight into the progenitor capacity of *Pi16*+ fibroblasts. However, until comprehensive lineage tracing experiments using genetic tools is be conducted, the concept of a step-wise lineage relationship in which all fibroblasts across all tissues develop from *Pi16+*CD26+ cells cannot be confirmed.

Indeed, whether *Pi16* marks a lineage of fibroblasts at all has not yet been shown – it is possible that *Pi16* is a biomarker for fibroblasts that have seen TGF-β ^11^ or experienced shear-stress ^36^ ( Figure 1). Additionally, it is a possibility that *Pi16+* and *Co1l5a1+* fibroblasts act as independent progenitors to distinct fibroblast lineages, such as pro-fibrotic progenitors and adipocyte precursor cells as seen in adipose tissues. ^30^^,^^31^ Further, cells within the fibroblast lineage may not become terminally differentiated states and are instead all phenotypically and functionally plastic given the correct cues. ^37^^,^^38^ Indeed, in many of the studies examined, the two *Dpt+* fibroblast subsets ^4^ are the predominant and sometimes sole fibroblasts in the examined tissue and are therefore the only fibroblasts available to respond to injury.

Outside of potential progenitor roles, *Pi16+* fibroblasts have been implicated in immunity, either through the direct activation of lymphocytes in the tonsil or through infiltration of immune cells after injury, potentially through promotion of increased endothelial permeability. While *Pi16+* cells appear to be localized near vasculature in many of the examined tissues, their effects on endothelial cells have not been thoroughly explored outside of the brain.

As Pi realizes in Yann Martel’s novel the *Life of Pi*, human existence harbors complexity and interdependency. The same sentiments may apply to our understanding of the fibroblast lineage and the cells within it. Fibroblasts are heterogeneous, allowing for their large number of functions throughout the body in tissue- and context-specific manners. The role of fibroblasts in a variety of pathologies such as cancer and autoimmunity is only recently being appreciated and understanding how fibroblasts develop and function and how these activities contribute to disease will be crucial in the development of effective therapeutics. Understanding how the *Pi16+* fibroblast functions and its ability to act in a both stem-like and immunomodulating fashion is a key first step in this process.

## Data Availability

No data are associated with this article.
