# Supplementary material for: Life of Pi: Exploring functions of Pi16+ fibroblasts
Source: F1000Res. 2024 Jul 8;13:126. Originally published 2024 Feb 20. [Version 2] doi: 10.12688/f1000research.143511.2 (PMC11196929; doi:10.12688/f1000research.143511.2)
Supplement: Peer review history

# Reviewer report: Reviewer response for version 2

**DOI:** 10.5256/f1000research.168794.r300232

**Published:** 2024-08-05

**Authors:** Natalia Pikor

**Affiliations:** 1 Medical Research Center, Kantonsspital St.Gallen, St.Gallen, Switzerland

The reviewer is satisfied with the updated version. 

 Two minor comments:

 The authors may consider whether mural cells are non-fibroblastic cells (Section 2, paragraph 2).

 The reference relating to the activation of T cells co-cultured with Hic1+ fibroblasts in the muscle injury model may be mistaken (Section 3, paragraph 5).

Is the review written in accessible language?

Yes

Are all factual statements correct and adequately supported by citations?

Yes

Are the conclusions drawn appropriate in the context of the current research literature?

Yes

Is the topic of the review discussed comprehensively in the context of the current literature?

Yes

Reviewer Expertise:

Stromal cell immunobiology

I confirm that I have read this submission and believe that I have an appropriate level of expertise to confirm that it is of an acceptable scientific standard.

# Reviewer report: Reviewer response for version 1

**DOI:** 10.5256/f1000research.157184.r286893

**Published:** 2024-06-24

**Authors:** Dongsheng Jiang, Runyi Cao

**Affiliations:** 1 Precision Research Center for Refractory Diseases, Shanghai General Hospital (Ringgold ID: 12482), Shanghai, Shanghai, China

Buechler and colleagues have summarized two types of functional roles of Pi16+ cells through extensive scRNA-seq analysis, alongside in vitro and in vivo models in both physiological and pathological conditions. Below are some minor comments to help provide a more comprehensive profile of Pi16+ fibroblasts.

 In the introduction, the authors introduce the concept that a precursor fibroblast state exists, which develops into specialized fibroblast subsets or states based on context-specific signalling. Following this, the authors give a comprehensive summary of Pi16+ fibroblast subsets from different tissues, using various animal and disease models. The table 1 is very informative.

 In the subsequent lineage function section, the authors describe the precursor phenotypes of Pi16+ fibroblasts, and attempt to demonstrate that Pi16 is a marker that identifies the precursor fibroblast state previously introduced. Furthermore, by highlighting Phenotypic similarities after targeted cell depletion, the authors provide a link between Pi16+ and Hic1+ fibroblasts, and even with CD201+ or CD26 (Dpp4)+ fibroblasts. However to substantiate the equivalency of Hic1+ fibroblasts (or CD201+ or CD26+ fibroblasts) to Pi16+ fibroblasts, more evidence is needed.  This could include coexpression at the mRNA level from sequencing data or coexpression at the protein level shown by, for example, immunofluorescence. Reinforcing this evidence would strengthen the conceptual claim.

 In the next section, the authors elucidate the non-precursor roles of Pi16+ fibroblasts, particularly their functions in inflammation, fibrosis and scarring. On Page 5, in the last paragraph the following statements regarding EPFs are not fully correct: “The En1.cre allele first emerges in the skin at embryonic day 16.5 (E16.5). The cells, named En1 positive fibroblasts (EPFs), become the dominant fibroblast population by P30.” Please refer to the follow-up study described in Jiang et al., 2018, Nat Cell Biol, to revise these two sentences. Furthermore, the study by Mascharak et al., 2021, Science, is also worth mentioning. It is essential to point out that En1 is a lineage marker of fibroblasts. It is expressed only during a very short time window in early embryonic development (Jiang et al., 2018 [Ref -1]), or reactivated upon injury (Mascharak et al., 2021[Ref-2]). Additionally, the cited Reference 16 is a BioRxiv paper that has since been published in Nature (Correa-Gallegos et al., 2023 [Ref -3]). Please update this reference.

 Small grammatical errors: verbs are missing in the following three sentences.

 1. On page 4, Paragraph 3: “This elegant cell transfer system confirmed that Pi16+ cells can...”

 2. On page 4, Paragraph 6: “The deletion of Hic1 also had an activating effect on these cells, comparison...”

 3. On page 6, Paragraph 3: “If Pi16+ fibroblasts are required for extravasation and immune infiltration, therapeutic exploitation...”

 Small spelling mistakes

 1. On page 5, Paragraph 1: “..., showing that that Pi16+ fibroblasts ...”, please delete the redundant “that”.

 2). On page 7 (Conclusion), Paragraph 2: “Indeed, whether Pi16 marks a lineage of fibroblasts... or experienced sheer-stress (Figure 1).” “sheer” should be “shear”.

Is the review written in accessible language?

Yes

Are all factual statements correct and adequately supported by citations?

Yes

Are the conclusions drawn appropriate in the context of the current research literature?

Yes

Is the topic of the review discussed comprehensively in the context of the current literature?

Yes

Reviewer Expertise:

Fibroblast heterogeneity, Scarring and Fibrosis

We confirm that we have read this submission and believe that we have an appropriate level of expertise to confirm that it is of an acceptable scientific standard.

## References

[1] : Two succeeding fibroblastic lineages drive dermal development and the transition from regeneration to scarring. *Nat Cell Biol* .2018;20(4) : 10.1038/s41556-018-0073-8 422-431 10.1038/s41556-018-0073-8 29593327

[2] : Preventing Engrailed-1 activation in fibroblasts yields wound regeneration without scarring. *Science* .2021;372(6540) : 10.1126/science.aba2374 10.1126/science.aba2374 33888614 PMC9008875

[3] : CD201+ fascia progenitors choreograph injury repair. *Nature* .2023;623(7988) : 10.1038/s41586-023-06725-x 792-802 10.1038/s41586-023-06725-x 37968392 PMC10665192

# Reviewer report: Reviewer response for version 1

**DOI:** 10.5256/f1000research.157184.r264918

**Published:** 2024-05-13

**Authors:** Natalia Pikor

**Affiliations:** 1 Medical Research Center, Kantonsspital St.Gallen, St.Gallen, Switzerland

Buechler and colleagues have prepared a comprehensive review of the many tissue settings and experimental models in which Pi16+ cells (or their transcriptionally similar counterparts) are demonstrated to respond to biological perturbation. There are minor comments that would be recommended to increase the clarity between lineage and non-lineage functions of Pi16+ fibroblasts. 

 In section 2., the authors describe experiments in which Pi16+ fibroblasts or their counterparts have been shown to serve as progenitors for more specialized fibroblastic cell types in vitro or using lineage-tracing models. Collectively the cited literature discusses the role for Pi16+ fibroblasts to give rise to adipocytes or to ECM-producing or contractile fibroblasts (in epicardial fibrosis or as myofibroblastic cancer associated fibroblasts in tumors). However, some of the studies quoted in the non-lineage functions of Pi16+ fibroblasts section appear to reflect a progenitor phenotype of Pi16+ or Pi16+-like cells. This includes the cited work by Rinkevich and colleagues, as well as pro-fibrotic function of fate-mapped 'mural chaser' cells in the adipose tissue. If one assumes that fibroblasts that give rise to CAFs with an ECM-enriched gene signature represent a progenitor-progeny relationship, is this not also the case for fibroblasts that differentiate to produce ECM and cover a scar in a tissue injury model?  

 What appears to stand out are Pi16+ cells in lymphoid organs. Unlike the perivascular progenitors that receive Lymphotoxin signals to differentiate into fibroblastic reticular cells (Cheng HW et al., 2019 Nat. Comm. (Ref 1 )and Prados A. et al., 2022, Nat. Imm (Ref 2)), Pi16+ reticular cells do not appear to be FRC progenitors (Pezoldt et al. 2018 Nat. Comm) [Ref4]. Nevertheless, these cells are expanded in inflammation, and encode a stemness gene signature, as shown in chronically-inflamed tonsils (De Martin et al., 2023 Nat. Immunol. (Ref 3)).  Perhaps, it is the case that they are either more inflammatory, as the authors have discussed, or respond according to the nature of the tissue injury. 

 Considering the above mentioned points, the authors may consider to reorganize these sections, and order the sections reflecting the graphical representation in Figure 1.   

 Figure 1 may be clarified. Fibroblasts activated by inflammation or injury may be those that recruit or activate immune cells (as in lymphoid organs) or exhibit an ECM-depositing phenotype (wounding or CAFs), respectively.  

 Minor comments: 

 Figure 1: It would be helpful to cite the relevant references related to the 4 feature of Pi16+ function.  

 The authors may consider discussing mural cells as fibroblasts. At present there is a discrepancy between non-fibroblastic mural cells – relating to reference 20 and adipocyte progenitors and Pi16+ fibroblasts derived from Pdgfrb+ mural cells – relating to reference 29. Are vascular smooth muscle cells and pericytes fundamentally not a fibroblastic cell type? After all,  adipocytes are included as specialized fibroblasts.  

 Reference 16 is mis-cited.  

 In reference 27 – it is not clear which CD34+ exhibits the Pi16+ phenotype 

 It is likely that the reticulated interstitium, like the fascia, is highly vascularized. Thus, although not mural cells, Pi16+ cells in these tissues may also be considered in proximity to the vasculature. 

 Small grammatical typos:  

 Section 1, Paragraph 1 of the introduction: 'fibroblasts that arise during inflammation are seeded from local, in situ local precursors …' 

 Section 1, Paragraph 4: 'Pi16+ fibroblasts have been identified near the vasculature in numerous tissues...'

Is the review written in accessible language?

Yes

Are all factual statements correct and adequately supported by citations?

Yes

Are the conclusions drawn appropriate in the context of the current research literature?

Yes

Is the topic of the review discussed comprehensively in the context of the current literature?

Yes

Reviewer Expertise:

Stromal cell immunobiology

I confirm that I have read this submission and believe that I have an appropriate level of expertise to confirm that it is of an acceptable scientific standard.

## References

[1] : Origin and differentiation trajectories of fibroblastic reticular cells in the splenic white pulp. *Nat Commun* .2019;10(1) : 10.1038/s41467-019-09728-3 1739 10.1038/s41467-019-09728-3 30988302 PMC6465367

[2] : Fibroblastic reticular cell lineage convergence in Peyer's patches governs intestinal immunity. *Nat Immunol* .2021;22(4) : 10.1038/s41590-021-00894-5 510-519 10.1038/s41590-021-00894-5 33707780 PMC7610542

[3] : PI16+ reticular cells in human palatine tonsils govern T cell activity in distinct subepithelial niches. *Nat Immunol* .2023;24(7) : 10.1038/s41590-023-01502-4 1138-1148 10.1038/s41590-023-01502-4 37202490 PMC10307632

[4] : Neonatally imprinted stromal cell subsets induce tolerogenic dendritic cells in mesenteric lymph nodes. *Nat Commun* .2018;9(1) : 10.1038/s41467-018-06423-7 3903 10.1038/s41467-018-06423-7 30254319 PMC6156403
